# Supplementary material for: Chronic Pain in Spanish Wildland Firefighters
Source: J Clin Med. 2022 Feb 14;11(4):989. doi: 10.3390/jcm11040989 (PMC8875785; doi:10.3390/jcm11040989)
Supplement: Supplementary file 1 [file jcm-11-00989-s001.zip › jcm-1564473-supplementary.pdf]

ANÁLISIS DESCRIPTIVO DE LAS PRINCIPALES LESIONES DE LAS BRIGADAS DE REFUERZO CONTRA INCENDIOS FORESTALES (BRIF)

A grandes rasgos, la labor de extinción de Incendios Forestales se constata como una labor eminentemente física muy exigente, dónde la larga duración del esfuerzo, condiciones climáticas hostiles y cambiantes, y la larga duración del esfuerzo conlleva al trabajador a una carga física muy elevada. En otras profesiones físicas tipo Bomberos de Estructura y Militares, se han descrito las principales lesiones y el riesgo potencial de sufrirlas en numerosos estudios, pero no es el caso del Bomberos Forestal. Por lo que el presente cuestionario, creado en conjunto por miembros de la Universidad de Deusto-Donosti (patxi.leon@deusto.es), y de la Universidad de León (fgarh@unileon.es), y con el apoyo de la Asociación de Trabajadores BRIF (AT-BRIF), tiene el objetivo de conocer y poder profundizar en la tipología de las lesiones y el dolor crónico de los Bomberos Forestales BRIF.

El cuestionario consta de 5 partes:
1ª Parte: variables sociodemográficas (personales y laborales)
2ª Parte: actividad física realizada en su vida diaria
3ª Parte: lesiones producidas dentro del ámbito laboral
4ª Parte: dolor crónico

La participación es totalmente voluntaria y anónima.

El tratamiento, la comunicación y la cesión de los datos de carácter personal de todos los sujetos participantes se ajustará a lo dispuesto en la Ley Orgánica 3/2018, de 5 de diciembre, de Protección de Datos Personales y garantía de los derechos digitales. De acuerdo a lo que establece la legislación mencionada, usted puede ejercer los derechos de oposición y cancelación de datos, para lo cual deberá dirigirse a los directores del estudio. Los investigadores del proyecto podrán tener acceso a los datos del voluntario. Los datos personales y la información obtenida de este estudio, con garantía de privacidad para su identidad, se conocerá sólo por los investigadores del proyecto. Con la aceptación de este consentimiento usted autoriza la recogida, almacenamiento y análisis de sus datos solicitados, desvinculados de la identidad por un sistema de codificación doble reversible. Aquellas personas que continúen el proceso mostrarán su aceptación a las características de la investigación y asegurará su comprensión.

*Obligatorio

1. 1.- He leído y acepto las condiciones: *

Marca solo un óvalo.

- Sí
- No

VARIABLES SOCIODEMOGRÁFICAS

En este apartado se preguntan cuestiones acerca de las características personales y laborales de los participantes, con el fin de contextualizar la muestra de una forma objetiva

2. 2.- Edad: *

3. 3.- Sexo: *

Marca solo un óvalo.

- Mujer
- Hombre
- Otro

4. 4.- Peso en Kg: *

5. 5.- Estatura en cm: *

6. 6.- ¿Eres fumador? *

Marca solo un óvalo.

- No
- Sí

7. 6.1- En el caso de ser fumador. ¿Cuántos cigarrillos fumas un día normal?

Marca solo un óvalo.

- 3 o menos
- De 4 a 10
- De 11 a 15
- 16 o más

8. 7.- ¿ Consumes alcohol? *

Marca solo un óvalo.

- No
- Sí, de forma ocasional (fin de semana)
- Sí, de forma habitual (a diario)

9. 8.- ¿ Has pasado la COVID- 2019? *

Marca solo un óvalo.

- No
- No lo sé con certeza
- Sí, asintomático
- Sí, síntomas leves
- Sí, síntomas graves sin hospitalización
- Sí, síntomas graves con hospitalización

10. 8.1.- En el caso de haber pasado la enfermedad de la COVID- 19, señala cuándo:

Marca solo un óvalo.

- Marzo 2020
- Abril 2020
- Mayo 2020
- Junio 2020
- Julio 2020
- Agosto 2020
- Septiembre 2020
- Octubre 2020
- Noviembre 2020
- Diciembre 2020
- Enero 2021
- Febrero 2021

11. 9.- Años de experiencia dentro del dispositivo BRIF: *

12. 10.- BRIF en la que trabajas actualmente (en el caso de ser destinado este año a Ruente, señala en la que tienes destino habitual + la pestaña de Ruente): *

Selecciona todos los que correspondan.

- [ ] Tineo
- [ ] Laza
- [ ] Lubia
- [ ] Puerto del Pico
- [ ] Tabuyo del monte
- [ ] Daroca
- [ ] Pinofranqueado
- [ ] La Iglesuela
- [ ] Prado de los Esquiladores
- [ ] Puntagorda
- [ ] Ruente

13. 11.- Puesto actual: *

Marca solo un óvalo.

- ( ) Especialista bombero/a forestal
- ( ) Capataz bombero/a forestal
- ( ) Técnico/a bombero/a forestal

14. 12.- Aproximadamente, peso del EPI + complementos utilizados (en kg) (ej: cantimplora, material de avituallamiento, ropa de cambio, etc.,):

____

15. 13.- Principales herramientas utilizadas durante la campaña de extinción: (señala las 2 más utilizadas) *

Selecciona todos los que correspondan.

- [ ] Mochila extintora
- [ ] Batefuegos
- [ ] Mochila extintora + batefuegos
- [ ] Pulaski, azada, gorgui, mcleod o similar
- [ ] Calabozo, tajamatas o similar
- [ ] Motosierra, cargas de la motosierra, etc.
- [ ] Herramienta rotatoria en función de cuadrante
- [ ] Carga de material, alimentos, avituallamiento, etc.
- [ ] Labores de gestión, control y/o organización

Otro: [ ] ______________________________

16. 14.- Principales herramientas/labores realizadas durante fase de labores preventivas (invierno): *

Selecciona todos los que correspondan.

- [ ] Desbrozadora
- [ ] Motosierra
- [ ] Otras herramientas mecánicas
- [ ] Labores de gestión, organización y/o control
- [ ] Acarreo de ramas o material

Otro: [ ] ____________________

17. 15.- En el caso de participar en quemas prescritas, y/o EPRIF indica las principales herramientas /acciones utilizadas/realizadas:

Selecciona todos los que correspondan.

- [ ] Antorcha de goteo
- [ ] Mochila extintora
- [ ] Batefuego
- [ ] Desbrozadora
- [ ] Motosierra
- [ ] Acarreo de ramas o material
- [ ] Rotación de funciones/ herramientas según cuadrante
- [ ] Labores de gestión, control y/o organización

Otro: [ ] ____________________

ACTIVIDAD FÍSICA

En este apartado se van a preguntar cuestiones acerca de la actividad física realizada durante tu día a día.

18. 16.- ¿Cuántas horas de media entrenas a la semana? *

Marca solo un óvalo.

- ( ) <1
- ( ) 1-3
- ( ) 3-5
- ( ) 5-7
- ( ) >7

19. 17.- Normalmente, en una semana típica: ¿cuántos días a la semana eres físicamente activo durante al menos 60 minutos? (La actividad física es cualquier actividad que incrementa tu ritmo cardíaco y te hace estar falto de aire en algún momento. Esta actividad física puede ser realizada en cualquier deporte, jugando con amistades, o caminando al trabajo) *

Marca solo un óvalo.

- Ningún día
- Un día
- Dos días
- Tres días
- Cuatro días
- Cinco días
- Seis días
- Siete días

20. 18. -¿Qué tipo de actividad física realizas? *

Selecciona todos los que correspondan.

- [ ] Caminata
- [ ] Senderismo
- [ ] Carrera
- [ ] Entrenamieto de fuerza
- [ ] Deportes de equipo (ej: rugby, fútbol, etc.)
- [ ] Deporte individual (ej: tenis, piragüismo, etc.)

Otro: [ ] ____________________

21. 18.1- ¿Realizas ejercicios específicos para el fortalecimiento del CORE? *

Marca solo un óvalo.

- No
- Sí

22. 19.- ¿Piensas que eres una persona activa comparada con otra gente de su edad? *

Marca solo un óvalo.

- Muy inactiva
- Inactiva
- Neutra
- Activa
- Muy activa

23. 20.- ¿Cómo describes tu salud en general? *

Marca solo un óvalo.

- Muy débil
- Débil
- Neutra
- Buena
- Muy buena

24. 20.1- De manera habitual, la calidad de tu sueño fue: *

Marca solo un óvalo.

- Muy mala
- Mala
- Pasable
- Buena
- Muy buena

3ª PARTE: LESIONES OCUPACIONALES

Primeramente y atendiendo a lo descrito por Phelps et al, (2018), "Una lesión se considera relacionada con el trabajo (ocupacional) si un evento o exposición en el ambiente de trabajo causó o contribuyó a la lesión o agravó significativamente una lesión ya existente". Así mismo se entiende que la lesión no implica directamente baja laboral, sino que puede existir o no, pero en todos los casos produce en el trabajador molestias y/o dolor, condicionando la calidad de vida y laboral del mismo (Scherzer et al, 2005).

Dentro del ambiente del trabajo entendemos: incendios o emergencias (desplazamiento, actuación o regreso), entrenamiento físico en base, prácticas técnico-tácticas (marchas, manejo de herramientas, etc), labores preventivas, quemas prescritas, etc.

En este apartado encontrarás distintas preguntas relacionadas con las lesiones producidas en el ámbito laboral.

25. 21.- ¿Has tenido alguna lesión relacionada con el trabajo? *

Marca solo un óvalo.

- No *Salta a la pregunta 97*
- Sí

LESIÓN 1

26. 22.- En el caso de haber sufrido alguna lesión relacionada con el trabajo, ¿Cuándo tuvo lugar la lesión?: *

Marca solo un óvalo.

- Durante el último año (entre febrero 2020 y el momento actual)
- Hace más de un año

27. 23.- Si la lesión fue en el último año, indica el mes en el que ocurrió:

Marca solo un óvalo.

- Febrero 2020
- Marzo 2020
- Abril 2020
- Mayo 2020
- Junio 2020
- Julio 2020
- Agosto 2020
- Septiembre 2020
- Octubre 2020
- Noviembre 2020
- Diciembre 2020
- Enero 2021
- Febrero 2021

28. 24.- Puesto de trabajo que ocupabas cuando se produjo la lesión: *

Marca solo un óvalo.

- Especialista Bombero/a Forestal
- Capataz Bombero/a Forestal
- Técnico/a Bombero/a Forestal

29. 25.- ¿Qué tipo de lesión fue? *

Marca solo un óvalo.

- Nueva lesión
- Recaída

30. 26.-Actividad que estabas realizando cuando ocurrió la lesión: *

Marca solo un óvalo.

- Incendio Forestal
- Otras emergencias
- Prácticas en base
- Entrenamiento físico en base
- Labores preventivas
- Otro: ______________________

31. 26.1.- En el caso de haber sufrido la lesión durante un "Incendio Forestal", señala la labor realizada en el momento de la misma:

Marca solo un óvalo.

- Aproximación
- Ataque directo
- Ataque Indirecto
- Mixto
- Vigilancia
- Labores de perimetración, liquidación
- Retirada
- No recordado
- Otro: ____________________

32. 26.2.-En el caso de haber sufrido la lesión durante "prácticas en base" señala la labor realizada en el momento de la misma:

Marca solo un óvalo.

- Manejo de herramientas (línea de defensa, practica de ataque directo, etc.)
- Marchas con EPI
- Marchas con EPI + herramienta
- Adecuación de material (limpieza, afilado, etc)
- Embarques y desembarques
- No recordado
- Otro: ____________________

33. 26.3.-En el caso de haber sufrido la lesión durante "entrenamiento físico en base" señala el tipo de entrenamiento realizado en el momento de la misma:

Marca solo un óvalo.

- Entrenamiento de fuerza
- Entrenamiento de resistencia
- Entrenamiento de flexibilidad/movilidad
- No recordado
- Otro: ____________________

34. 26.4.-En el caso de haber sufrido la lesión durante "labores preventivas" señala el tipo de tarea o labor realizada en el momento de la misma:

Marca solo un óvalo.

- Desbroce
- Quemas prescritas
- Acceso
- Transporte de cargas
- Acarreo de material y ramas
- Quemas de restos de material
- No recordado
- Otro: ____________________

35. 27.- Causa principal de la lesión: *

Marca solo un óvalo.

- Impacto
- Sobreuso
- Desconocida
- Otro: ____________________

36. 27.1.- En el caso de haber seleccionado la opción IMPACTO, señala la opción que ocasionó la lesión:

Marca solo un óvalo.

- Caída
- Salto
- Resbalón
- Tropiezo
- Golpeo de un objeto
- Otro: ____________________

37. 27.2.- En el caso de haber seleccionado la opción SOBREUSO, señala la opción que ocasionó la lesión:

Marca solo un óvalo.

- Esfuerzo excesivo
- Tareas repetitivas
- Exposición a las llamas
- Clima extremo (frío o calor)
- Otro: ____________________

38. 28.- Tipo de lesión: *

Marca solo un óvalo.

- Quemadura
- Insolación
- Inhalación de humos
- Dolor muscular
- Tendinitis
- Rotura muscular
- Esguince osteo-articular
- Dislocación articular
- Fisura ósea
- Fractura
- Herida/corte, con sangrado abundante
- Conmoción cerebral
- Golpe
- Ampollas
- Otro: ____________________

39. 29.- Parte del cuerpo lesionada: *

Marca solo un óvalo.

- Cabeza
- Cuello
- Nariz
- Ojos
- Oídos
- Dedos- mano
- Mano
- Muñeca
- Antebrazo
- Codo
- Hombro
- Clavícula
- Espalda alta (zona cervical)
- Espalda media (zona torácica)
- Espalda baja (zona lumbar)
- Costillas
- Pecho
- Abdomen
- Cadera
- Pelvis
- Muslo
- Rodilla
- Pierna
- Tobillo
- Pie
- Uñas- pie
- Dedos píe
- Fascia plantar
- Talón
- Otro: ____________________

40. 30.-Duración de la lesión: *

Marca solo un óvalo.

- < 1 semana
- 1-3 semanas
- 3-6 semanas
- > 6 semanas

41. 31.- La lesión requirió atención médica: *

Marca solo un óvalo.

- No
- Sí

42. 32.- ¿La lesión requirió baja o ausencia al puesto de trabajo? *

Marca solo un óvalo.

- No
- Sí

43. ¿Has tenido más lesiones en el trabajo?

Marca solo un óvalo.

- No *Salta a la pregunta 97*
- Sí

LESIÓN 2

44. 22.- En el caso de haber sufrido alguna lesión relacionada con el trabajo, ¿Cuándo tuvo lugar la lesión?: *

Marca solo un óvalo.

- Durante el último año (entre febrero 2020 y el momento actual)
- Hace más de un año

45. 23.- Si la lesión fue en el último año, indica el mes en el que ocurrió:

Marca solo un óvalo.

- Febrero 2020
- Marzo 2020
- Abril 2020
- Mayo 2020
- Junio 2020
- Julio 2020
- Agosto 2020
- Septiembre 2020
- Octubre 2020
- Noviembre 2020
- Diciembre 2020
- Enero 2021
- Febrero 2021

46. 24.- Puesto de trabajo que ocupabas cuando se produjo la lesión: *

Marca solo un óvalo.

- Especialista Bombero/a Forestal
- Capataz Bombero/a Forestal
- Técnico/a Bombero/a Forestal

47. 25.- ¿Qué tipo de lesión fue? *

Marca solo un óvalo.

- Nueva lesión
- Recaída

48. 26.-Actividad que estabas realizando cuando ocurrió la lesión: *

Marca solo un óvalo.

- Incendio Forestal
- Otras emergencias
- Prácticas en base
- Entrenamiento físico en base
- Labores preventivas
- Otro: ____________________

49. 26.1.- En el caso de haber sufrido la lesión durante un "Incendio Forestal", señala la labor realizada en el momento de la misma:

Marca solo un óvalo.

- Aproximación
- Ataque directo
- Ataque Indirecto
- Mixto
- Vigilancia
- Labores de perimetración, liquidación
- Retirada
- No recordado
- Otro: ________________

50. 26.2.-En el caso de haber sufrido la lesión durante "prácticas en base" señala la labor realizada en el momento de la misma:

Marca solo un óvalo.

- Manejo de herramientas (línea de defensa, practica de ataque directo, etc.)
- Marchas con EPI
- Marchas con EPI + herramienta
- Adecuación de material (limpieza, afilado, etc)
- Embarques y desembarques
- No recordado
- Otro: ________________

51. 26.3.-En el caso de haber sufrido la lesión durante "entrenamiento físico en base" señala el tipo de entrenamiento realizado en el momento de la misma:

Marca solo un óvalo.

- Entrenamiento de fuerza
- Entrenamiento de resistencia
- Entrenamiento de flexibilidad/movilidad
- No recordado
- Otro: ________________

52. 26.4.-En el caso de haber sufrido la lesión durante "labores preventivas" señala el tipo de tarea o labor realizada en el momento de la misma:

Marca solo un óvalo.

- Desbroce
- Quemas prescritas
- Acceso
- Transporte de cargas
- Acarreo de material y ramas
- Quemas de restos de material
- No recordado
- Otro: ______________________

53. 27.- Causa principal de la lesión: *

Marca solo un óvalo.

- Impacto
- Sobreuso
- Desconocida
- Otro: ______________________

54. 27.1.- En el caso de haber seleccionado la opción IMPACTO, señala la opción que ocasionó la lesión:

Marca solo un óvalo.

- Caída
- Salto
- Resbalón
- Tropiezo
- Golpeo de un objeto
- Otro: ______________________

55. 27.2.- En el caso de haber seleccionado la opción SOBREUSO, señala la opción que ocasionó la lesión:

Marca solo un óvalo.

- Esfuerzo excesivo
- Tareas repetitivas
- Exposición a las llamas
- Clima extremo (frío o calor)
- Otro: ______________________

56. 28.- Tipo de lesión: *

Marca solo un óvalo.

- Quemadura
- Insolación
- Inhalación de humos
- Dolor muscular
- Tendinitis
- Rotura muscular
- Esguince osteo-articular
- Dislocación articular
- Fisura ósea
- Fractura
- Herida/corte, con sangrado abundante
- Conmoción cerebral
- Golpe
- Ampollas
- Otro: ______________________

57. 29.- Parte del cuerpo lesionada: *

Marca solo un óvalo.

- Cabeza
- Cuello
- Nariz
- Ojos
- Oídos
- Dedos- mano
- Mano
- Muñeca
- Antebrazo
- Codo
- Hombro
- Clavícula
- Espalda alta (zona cervical)
- Espalda media (zona torácica)
- Espalda baja (zona lumbar)
- Costillas
- Pecho
- Abdomen
- Cadera
- Pelvis
- Muslo
- Rodilla
- Pierna
- Tobillo
- Pie
- Uñas- pie
- Dedos píe
- Fascia plantar
- Talón
- Otro: ______________________

58. 30.-Duración de la lesión: *

Marca solo un óvalo.

- < 1 semana
- 1-3 semanas
- 3-6 semanas
- > 6 semanas

59. 31.- La lesión requirió atención médica: *

Marca solo un óvalo.

- No
- Sí

60. 32.- ¿La lesión requirió baja o ausencia al puesto de trabajo? *

Marca solo un óvalo.

- No
- Sí

61. ¿Has tenido más lesiones en el trabajo?

Marca solo un óvalo.

- No *Salta a la pregunta 97*
- Sí

LESIÓN 3

62. 22.- En el caso de haber sufrido alguna lesión relacionada con el trabajo, ¿Cuándo tuvo lugar la lesión?: *

Marca solo un óvalo.

- Durante el último año (entre febrero 2020 y el momento actual)
- Hace más de un año

63. 23.- Si la lesión fue en el último año, indica el mes en el que ocurrió:

Marca solo un óvalo.

- Febrero 2020
- Marzo 2020
- Abril 2020
- Mayo 2020
- Junio 2020
- Julio 2020
- Agosto 2020
- Septiembre 2020
- Octubre 2020
- Noviembre 2020
- Diciembre 2020
- Enero 2021
- Febrero 2021

64. 24.- Puesto de trabajo que ocupabas cuando se produjo la lesión: *

Marca solo un óvalo.

- Especialista Bombero/a Forestal
- Capataz Bombero/a Forestal
- Técnico/a Bombero/a Forestal

65. 25.- ¿Qué tipo de lesión fue? *

Marca solo un óvalo.

- Nueva lesión
- Recaída

66. 26.-Actividad que estabas realizando cuando ocurrió la lesión: *

Marca solo un óvalo.

- Incendio Forestal
- Otras emergencias
- Prácticas en base
- Entrenamiento físico en base
- Labores preventivas
- Otro: ____________________

67. 26.1.- En el caso de haber sufrido la lesión durante un "Incendio Forestal", señala la labor realizada en el momento de la misma:

Marca solo un óvalo.

- Aproximación
- Ataque directo
- Ataque Indirecto
- Mixto
- Vigilancia
- Labores de perimetración, liquidación
- Retirada
- No recordado
- Otro: __________

68. 26.2.-En el caso de haber sufrido la lesión durante "prácticas en base" señala la labor realizada en el momento de la misma:

Marca solo un óvalo.

- Manejo de herramientas (línea de defensa, practica de ataque directo, etc.)
- Marchas con EPI
- Marchas con EPI + herramienta
- Adecuación de material (limpieza, afilado, etc)
- Embarques y desembarques
- No recordado
- Otro: __________

69. 26.3.-En el caso de haber sufrido la lesión durante "entrenamiento físico en base" señala el tipo de entrenamiento realizado en el momento de la misma:

Marca solo un óvalo.

- Entrenamiento de fuerza
- Entrenamiento de resistencia
- Entrenamiento de flexibilidad/movilidad
- No recordado
- Otro: __________

70. 26.4.-En el caso de haber sufrido la lesión durante "labores preventivas" señala el tipo de tarea o labor realizada en el momento de la misma:

Marca solo un óvalo.

- Desbroce
- Quemas prescritas
- Acceso
- Transporte de cargas
- Acarreo de material y ramas
- Quemas de restos de material
- No recordado
- Otro: ______________________

71. 27.- Causa principal de la lesión: *

Marca solo un óvalo.

- Impacto
- Sobreuso
- Desconocida
- Otro: ______________________

72. 27.1.- En el caso de haber seleccionado la opción IMPACTO, señala la opción que ocasionó la lesión:

Marca solo un óvalo.

- Caída
- Salto
- Resbalón
- Tropiezo
- Golpeo de un objeto
- Otro: ______________________

73. 27.2.- En el caso de haber seleccionado la opción SOBREUSO, señala la opción que ocasionó la lesión:

Marca solo un óvalo.

- Esfuerzo excesivo
- Tareas repetitivas
- Exposición a las llamas
- Clima extremo (frío o calor)
- Otro: ______________________

74. 28.- Tipo de lesión: *

Marca solo un óvalo.

- Quemadura
- Insolación
- Inhalación de humos
- Dolor muscular
- Tendinitis
- Rotura muscular
- Esguince osteo-articular
- Dislocación articular
- Fisura ósea
- Fractura
- Herida/corte, con sangrado abundante
- Conmoción cerebral
- Golpe
- Ampollas
- Otro: ______________________

75. 29.- Parte del cuerpo lesionada: *

Marca solo un óvalo.

- Cabeza
- Cuello
- Nariz
- Ojos
- Oídos
- Dedos- mano
- Mano
- Muñeca
- Antebrazo
- Codo
- Hombro
- Clavícula
- Espalda alta (zona cervical)
- Espalda media (zona torácica)
- Espalda baja (zona lumbar)
- Costillas
- Pecho
- Abdomen
- Cadera
- Pelvis
- Muslo
- Rodilla
- Pierna
- Tobillo
- Pie
- Uñas- pie
- Dedos píe
- Fascia plantar
- Talón
- Otro: ______________________

76. 30.-Duración de la lesión: *

Marca solo un óvalo.

- < 1 semana
- 1-3 semanas
- 3-6 semanas
- > 6 semanas

77. 31.- La lesión requirió atención médica: *

Marca solo un óvalo.

- No
- Sí

78. 32.- ¿La lesión requirió baja o ausencia al puesto de trabajo? *

Marca solo un óvalo.

- No
- Sí

79. ¿Has tenido más lesiones en el trabajo?

Marca solo un óvalo.

- No *Salta a la pregunta 97*
- Sí

LESIÓN 4

80. 22.- En el caso de haber sufrido alguna lesión relacionada con el trabajo, ¿Cuándo tuvo lugar la lesión?: *

Marca solo un óvalo.

- Durante el último año (entre febrero 2020 y el momento actual)
- Hace más de un año

81. 23.- Si la lesión fue en el último año, indica el mes en el que ocurrió:

Marca solo un óvalo.

- Febrero 2020
- Marzo 2020
- Abril 2020
- Mayo 2020
- Junio 2020
- Julio 2020
- Agosto 2020
- Septiembre 2020
- Octubre 2020
- Noviembre 2020
- Diciembre 2020
- Enero 2021
- Febrero 2021

82. 24.- Puesto de trabajo que ocupabas cuando se produjo la lesión: *

Marca solo un óvalo.

- Especialista Bombero/a Forestal
- Capataz Bombero/a Forestal
- Técnico/a Bombero/a Forestal

83. 25.- ¿Qué tipo de lesión fue? *

Marca solo un óvalo.

- Nueva lesión
- Recaída

84. 26.-Actividad que estabas realizando cuando ocurrió la lesión: *

Marca solo un óvalo.

- Incendio Forestal
- Otras emergencias
- Prácticas en base
- Entrenamiento físico en base
- Labores preventivas
- Otro: ____________

85. 26.1.- En el caso de haber sufrido la lesión durante un "Incendio Forestal", señala la labor realizada en el momento de la misma:

Marca solo un óvalo.

- Aproximación
- Ataque directo
- Ataque Indirecto
- Mixto
- Vigilancia
- Labores de perimetración, liquidación
- Retirada
- No recordado
- Otro: ____________________

86. 26.2.-En el caso de haber sufrido la lesión durante "prácticas en base" señala la labor realizada en el momento de la misma:

Marca solo un óvalo.

- Manejo de herramientas (línea de defensa, practica de ataque directo, etc.)
- Marchas con EPI
- Marchas con EPI + herramienta
- Adecuación de material (limpieza, afilado, etc)
- Embarques y desembarques
- No recordado
- Otro: ____________________

87. 26.3.-En el caso de haber sufrido la lesión durante "entrenamiento físico en base" señala el tipo de entrenamiento realizado en el momento de la misma:

Marca solo un óvalo.

- Entrenamiento de fuerza
- Entrenamiento de resistencia
- Entrenamiento de flexibilidad/movilidad
- No recordado
- Otro: ____________________

88. 26.4.-En el caso de haber sufrido la lesión durante "labores preventivas" señala el tipo de tarea o labor realizada en el momento de la misma:

Marca solo un óvalo.

- Desbroce
- Quemas prescritas
- Acceso
- Transporte de cargas
- Acarreo de material y ramas
- Quemas de restos de material
- No recordado
- Otro: ______________________

89. 27.- Causa principal de la lesión: *

Marca solo un óvalo.

- Impacto
- Sobreuso
- Desconocida
- Otro: ______________________

90. 27.1.- En el caso de haber seleccionado la opción IMPACTO, señala la opción que ocasionó la lesión:

Marca solo un óvalo.

- Caída
- Salto
- Resbalón
- Tropiezo
- Golpeo de un objeto
- Otro: ______________________

91. 27.2.- En el caso de haber seleccionado la opción SOBREUSO, señala la opción que ocasionó la lesión:

Marca solo un óvalo.

- Esfuerzo excesivo
- Tareas repetitivas
- Exposición a las llamas
- Clima extremo (frío o calor)
- Otro: ______________________

92. 28.- Tipo de lesión: *

Marca solo un óvalo.

- Quemadura
- Insolación
- Inhalación de humos
- Dolor muscular
- Tendinitis
- Rotura muscular
- Esguince osteo-articular
- Dislocación articular
- Fisura ósea
- Fractura
- Herida/corte, con sangrado abundante
- Conmoción cerebral
- Golpe
- Ampollas
- Otro: ____________________

93. 29.- Parte del cuerpo lesionada: *

Marca solo un óvalo.

- Cabeza
- Cuello
- Nariz
- Ojos
- Oídos
- Dedos- mano
- Mano
- Muñeca
- Antebrazo
- Codo
- Hombro
- Clavícula
- Espalda alta (zona cervical)
- Espalda media (zona torácica)
- Espalda baja (zona lumbar)
- Costillas
- Pecho
- Abdomen
- Cadera
- Pelvis
- Muslo
- Rodilla
- Pierna
- Tobillo
- Pie
- Uñas- pie
- Dedos píe
- Fascia plantar
- Talón
- Otro: ____________________

94. 30.-Duración de la lesión: *

Marca solo un óvalo.

- < 1 semana
- 1-3 semanas
- 3-6 semanas
- > 6 semanas

95. 31.- La lesión requirió atención médica: *

Marca solo un óvalo.

- No
- Sí

96. 32.- ¿La lesión requirió baja o ausencia al puesto de trabajo? *

Marca solo un óvalo.

- No
- Sí

4ª PARTE: DOLOR CRÓNICO

Entendemos que el dolor crónico es un dolor de más de 3 meses de duración que puede resultar de una enfermedad, lesión, traumatismo, cirugía o de origen desconocido (Levins et al, 2019). En este apartado dispones de varias preguntas en relación al dolor crónico relacionado con el desarrollo de tu profesión.

97. 33- ¿Sufres dolor crónico o molestias crónicas relacionadas con el desarrollo de tu profesión? *

Marca solo un óvalo.

- No
- Sí

98. 33.1. Si has respondido si, indica donde:

Selecciona todos los que correspondan.

- [ ] Cabeza
- [ ] Cuello
- [ ] Nariz
- [ ] Ojos
- [ ] Oídos
- [ ] Dedos- mano
- [ ] Mano
- [ ] Muñeca
- [ ] Antebrazo
- [ ] Codo
- [ ] Hombro
- [ ] Clavícula
- [ ] Espalda alta (zona cervical)
- [ ] Espalda media (zona torácica)
- [ ] Espalda baja (zona lumbar)
- [ ] Costillas
- [ ] Pecho
- [ ] Abdomen
- [ ] Cadera
- [ ] Pelvis
- [ ] Muslo
- [ ] Rodilla
- [ ] Pierna
- [ ] Tobillo
- [ ] Pie
- [ ] Uñas- pie
- [ ] Dedos píe
- [ ] Fascia plantar
- [ ] Talón

Otro: [ ] ____________________

99. 33.2.- ¿A qué crees que se debe?

Selecciona todos los que correspondan.

- [ ] Mala preparación
- [ ] Es una vieja lesión
- [ ] Cargas de entrenamiento (demasiado entrenamiento)
- [ ] Cargas de entrenamiento (poco entrenamiento)
- [ ] Malas posturas laborales
- [ ] Mantenimiento deficiente del material

Otro: [ ] ____________________

AGRADECIMIENTOS

Agradecemos enormemente que hayas dedicado unos minutos de tu tiempo en rellenar el presente cuestionario. Del mismo modo agradeceríamos que compartas el cuestionario con compañeros de profesión BRIF, con el fin de conseguir una participación representativa, y poder establecer conclusiones válidas y objetivas.

100. Si estás interesado en recibir los resultados de esta investigación especifica tu CORREO ELECTRÓNICO a continuación

101. GRACIAS POR TU TIEMPO. Si tienes algún comentario o aclaración que desees realizar sobre el cuestionario utilice el siguiente espacio (con cualquier consulta no dude en contactarnos: fgarh@unileon.es o patxi.leon@deusto.es)

Este contenido no ha sido creado ni aprobado por Google.

Google Formularios
